# Effect of prophylactic subcutaneous unfractionated heparin on the maternal sFlt-1/PlGF ratio: a retrospective cohort study

**DOI:** 10.1186/s12884-026-09306-8

**Published:** 2026-05-22

**Authors:** Yuri Yoshida, Takayuki Iriyama, Yu Ariyoshi, Yuji Ida, Eriko Yano, Haruka Matsui, Kensuke Suzuki, Ayako Hashimoto, Mari Ichinose, Masatake Toshimitsu, Seisuke Sayama, Kenbun Sone, Osamu Wada-Hiraike, Keiichi Kumasawa, Miyuki Harada, Yasushi Hirota

**Affiliations:** Department of Obstetrics and Gynecology, Faculty of Medicine, University of Tokyo, 7-3-1 Hongo, Bunkyo- ku, Tokyo, 113-8655 Japan

**Keywords:** Heparin, Unfractionated heparin, sFlt-1, PlGF, Preeclampsia

## Abstract

**Background:**

The ratio of soluble fms-like tyrosine kinase-1 (sFlt-1) to placental growth factor (PlGF) is widely used to assess the risk of preeclampsia. However, heparin can transiently increase the level of circulating sFlt-1, making it unclear whether prophylactic subcutaneous unfractionated heparin (UFH)—commonly used for thromboprophylaxis in pregnancy—affects the reliability of this biomarker.

**Methods:**

We conducted a retrospective cohort study to compare mid-trimester (18w0d-23w6d) sFlt-1 levels and sFlt-1/PlGF ratios in 45 pregnant women receiving prophylactic subcutaneous UFH for antiphospholipid syndrome or a history of thrombosis and 563 controls. We also examined the association between biomarker levels and time elapsed since the last UFH injection.

**Results:**

Mid-trimester sFlt-1 levels (1900 vs. 1782 pg/mL, *p* = 0.15) and sFlt-1/PlGF ratios (7.2 vs. 6.7, *p* = 0.87) were equivalent between the UFH and control groups. Multivariate regression analysis adjusting for maternal age, body mass index, gestational age at sampling, and low-dose aspirin intake confirmed no significant association between prophylactic UFH and sFlt-1 levels or sFlt-1/PlGF ratios. Within the UFH group, no correlation was found between the time since administration (range 63–477 min) and sFlt-1 levels (*r*=-0.049, *p* = 0.75) or sFlt-1/PlGF ratios (*r* = 0.019, *p* = 0.90).

**Conclusion:**

Prophylactic subcutaneous UFH was not associated with significant differences in sFlt-1/PlGF ratios, and the serum levels were not correlated with the timing of administration within the examined window. These findings suggest that the sFlt-1/PlGF ratio may remain a reliable tool for preeclampsia risk assessment in women receiving UFH prophylaxis under routine clinical sampling conditions.

## Background

Preeclampsia (PE) is a major obstetric complication that seriously affects maternal and fetal prognosis [1–3]. In recent years, the role of angiogenic factors in its pathophysiology has drawn increasing attention, and the ratio of the soluble fms-like tyrosine kinase-1 (sFlt-1) to placental growth factor (PlGF) is used worldwide as a highly reliable marker for short-term prediction of PE onset, severity assessment, and risk stratification [1, 4–11].

Pregnant women with thrombophilia, such as those with antiphospholipid syndrome (APS) or a history of thrombosis, require prophylactic anticoagulation to prevent venous thromboembolism [12–17]. While low-molecular-weight heparin (LMWH) is widely recommended, prophylactic subcutaneous UFH remains a global standard of care. It has demonstrated comparable or superior efficacy to LMWH for preventing recurrent pregnancy loss in women with APS [12, 22–24] and plays a critical role in peripartum management as a reversible bridging therapy [25]. These women remain at elevated risk of PE even under appropriate anticoagulation [13, 17], making it particularly important to ensure assessment using the sFlt-1/PlGF ratio is accurate. However, the interpretation of this biomarker in such patients is complicated by reports that heparin formulations can transiently increase circulating sFlt-1 levels through displacement from heparan sulfate proteoglycans (HSPG) on the cell surface [18–21]. Indeed, subcutaneous low-molecular-weight heparin (LMWH) increases sFlt-1 concentrations by approximately 1.5-fold several hours after administration [22], and intravenous unfractionated heparin (UFH) can induce a rapid, marked rise that exceeds 50-fold [23, 24].

Although evidence exists regarding increases in sFlt-1 levels due to subcutaneous LMWH and intravenous UFH, no data are available on the effects of subcutaneous UFH, another widely used prophylactic anticoagulant in pregnancy in instances of APS-related recurrent pregnancy loss or as bridging therapy near delivery [12, 25, 26]. Importantly, UFH differs from LMWH in its heterogeneous molecular weight and low, variable bioavailability (~ 30%) after subcutaneous injection [27–29], suggesting that previously reported findings for LMWH or intravenous UFH cannot be directly extrapolated. Given this gap in evidence, the effect of prophylactic subcutaneous UFH on the maternal sFlt-1/PlGF ratio remains unclear, creating uncertainty in PE risk assessment for many high-risk pregnant women receiving this therapy. Therefore, this study aimed to determine whether prophylactic subcutaneous UFH administration affects the maternal sFlt-1/PlGF ratio.

## Materials and methods

### Study design and ethics

This retrospective cohort study was conducted at The University of Tokyo Hospital between July 2022 and October 2024 in accordance with the Declaration of Helsinki and with the approval of The University of Tokyo Institutional Review Board (Approval No: 3053-1). Clinical data were obtained from electronic medical records in accordance with the institution’s comprehensive consent policy and the opt-out procedure disclosed on the hospital website.

### Study population

The UFH group was comprised of 45 patients who had an indication for prophylactic anticoagulation, such as APS or a history of thrombosis, and had been receiving subcutaneous UFH at 10,000 IU (5,000 IU twice daily) or 5,000 IU (2,500 IU twice daily) from early pregnancy. The control group consisted of 563 pregnant women who delivered at the same hospital between July 2022 and June 2023 and did not receive UFH during pregnancy. Eligibility criteria for both groups required an outpatient visit between 18 weeks 0 days and 23 weeks 6 days of gestation. At our institution, serum sFlt-1 and PlGF concentrations are measured in all pregnant women during routine outpatient prenatal checkups for PE screening with patient consent, and these values were used in this study. Cases of multiple pregnancies, pregnancies with uterine anomalies, and cases where subcutaneous UFH was discontinued before 18 weeks 0 days were excluded.

### Data collection

In the UFH group, data on the indication for UFH, dosage, and administration times were collected from medical records. In both groups, maternal background factors (age, parity, body mass index [BMI], conception method, smoking history, chronic hypertension [CH], low-dose aspirin [LDA] intake, history of PE) and perinatal outcomes (gestational age at delivery, birth weight, incidence of PE) were collected from medical records. Additionally, serum sFlt-1 and PlGF concentrations measured between 18 weeks 0 days and 23 weeks 6 days, gestational age at collection, and time of collection were gathered.

BMI was calculated using self-reported pre-pregnancy weight or weight at the first visit up to 12 weeks of gestation. Gestational age was estimated based on the last menstrual period and early pregnancy ultrasound results. Preterm birth was defined as delivery between 22 weeks 0 days and 36 weeks 6 days. The diagnosis of Hypertensive Disorders of Pregnancy and classification of its subtypes, including CH, gestational hypertension, PE, and superimposed PE, were based on the diagnostic criteria of the International Society for the Study of Hypertension in Pregnancy [30].

### Biomarker measurement

Serum sFlt-1 and PlGF levels were measured by a fully automated electrochemiluminescence immunoassay on Elecsys^®^ cobas e analyzers (Roche Diagnostics, Mannheim, Germany). The intra- and inter-assay coefficients of variation were < 5% and < 10%, respectively. The detection ranges were 10–85,000 pg/mL for sFlt-1 and 3–10,000 pg/mL for PlGF. The sFlt-1/PlGF ratio was calculated from the measured concentrations. According to the manufacturer’s instructions, these assays are not significantly affected by common endogenous or exogenous interferences under standard testing conditions.

### Statistical analysis

Statistical analysis was performed using EZR (Easy R) ver. 1.62 [31]. Continuous variables were compared using the Mann–Whitney U test, and Fisher’s exact test was used for nominal variables. Spearman’s rank correlation coefficient was used for correlation analysis between variables. To assess the independent effect of UFH administration on biomarker levels, multivariate linear regression analysis was performed. The model included maternal age, BMI (> 25 kg/m²), gestational age at blood sampling, and LDA intake as covariates [32, 33]. A p-value < 0.05 was considered statistically significant. Subgroup analyses within the UFH group were performed using the Kruskal–Wallis test to compare serum sFlt-1, PlGF, and sFlt-1/PlGF ratio among patients with different clinical indications (APS, history of thrombosis, and recurrent/habitual miscarriage).

## Results

Among pregnant women who received perinatal management, 45 patients who received subcutaneous UFH met the eligibility criteria (UFH group). The control group consisted of 563 pregnant women. The main indications for UFH administration in the UFH group were APS in 25 (55.6%), history of thrombosis in 14 (31.1%), and recurrent/habitual miscarriage in 6 (13.3%) patients. The most common UFH dosage was 10,000 IU/day in 41 (91.1%), and 5,000 IU/day in 4 (8.9%) patients, all of whom were reduced due to side effects, such as liver dysfunction. All patients received subcutaneous administration twice daily, every 12 h. In the UFH group, subgroup analysis according to the indication for therapy (APS, history of thrombosis, and recurrent/habitual miscarriage) showed no significant differences in serum sFlt-1, PlGF, or the sFlt-1/PlGF ratio among the three groups.

The patient characteristics for the UFH and control groups are shown in Table 1. The two groups showed no significant differences regarding maternal age (*p* = 0.42), proportion of primiparous women (*p* = 0.22), proportion with BMI > 25 kg/m² (*p* = 0.81), pregnancy rate by assisted reproductive technology (*p* = 0.19), smoking history (*p* > 0.99), CH (*p* > 0.99), and history of PE (*p* = 0.11). However, the rate of LDA intake was significantly higher in the UFH group than in the control group (75.6% vs. 20.8%, *p* < 0.001).

**Table 1:** Patient characteristics. Comparison of patient characteristics between the UFH group (*n* = 45) and the control group (*n* = 563). Data are shown as median [Interquartile Range (IQR)] or n (%). Continuous variables were analyzed using the Mann-Whitney U test, and nominal variables using Fisher’s exact test. UFH, Unfractionated Heparin; ART, Assisted Reproductive Technology; CH, Chronic Hypertension; PE, Preeclampsia; LDA, Low-dose Aspirin

	UFH (*n* = 45)	Control (*n* = 563)	*P* value
Age (y)	36.0 (33.0–40.0)	36.0 (32.0–39.0)	0.42
Primiparity	23 (51.1%)	234 (41.6%)	0.22
Body mass index > 25 kg/m²	4 (8.9%)	67 (11.9%)	0.81
ART	19 (42.4%)	183 (32.5%)	0.19
Smoking History	1 (2.2%)	18 (3.2%)	> 0.99
CH	1 (2.2%)	25 (4.4%)	> 0.99
History of PE	3 (6.7%)	13 (2.3%)	0.11
LDA intake	34 (75.6%)	117 (20.8%)	< 0.001

A comparison of serum sFlt-1, PlGF, and sFlt-1/PlGF ratios measured between 18 weeks 0 days and 23 weeks 6 days is shown in Table 2. The UFH and control groups showed no statistically significant differences in gestational age at blood sampling (weeks+days: 19 + 2 [18 + 5–19 + 5] vs. 19 + 1 [18 + 5–19 + 5], *p* = 0.96), sFlt-1 concentration (pg/mL: 1900 [1465–2704] vs. 1782 [1273–2501], *p* = 0.15), PlGF concentration (pg/mL: 315 [198–396] vs. 267 [190–368], *p* = 0.087), or sFlt-1/PlGF ratio (7.2 [5.0–9.2] vs. 6.7 [4.6–9.7], *p* = 0.87).

**Table 2:** Comparison of sFlt-1, PlGF, and sFlt-1/PlGF ratio values. Comparison between the UFH (*n* = 45) and control (*n* = 563) groups. Data are shown as median (IQR). Mann-Whitney U test was used for statistical analysis. UFH, Unfractionated Heparin; sFlt-1, soluble fms-like tyrosine kinase-1; PlGF, Placental Growth Factor

	UFH (*n* = 45)	Control (*n* = 563)	*P* value
Gestational age at sFlt-1 sampling (Weeks^+days^)	19^+ 2^ (18^+ 5^–19^+ 5^)	19^+ 1^ (18^+ 5^–19^+ 5^)	0.96
sFlt-1 (pg/mL)	1900 (1465–2704)	1782 (1273–2501)	0.15
PlGF (pg/mL)	315 (198–396)	267 (190–368)	0.087
sFlt-1/PlGF	7.2 (5.0–9.2)	6.7 (4.6–9.7)	0.87

We further performed multivariate linear regression analysis to adjust for potential confounders, including maternal age, BMI (> 25 kg/m²), gestational age at sampling, and LDA intake. The analysis demonstrated that prophylactic UFH administration was not significantly associated with serum sFlt-1 levels (coefficient 222.5, 95% confidence interval (CI) -128.5 to 573.4, *p* = 0.21), PlGF levels (coefficient 31.4, 95% CI -15.8 to 78.5, *p* = 0.19), or the sFlt-1/PlGF ratio (coefficient − 0.70, 95% CI -3.82 to 2.42, *p* = 0.66) (Table 3).

**Table 3:** Multivariate linear regression analysis of the effect of prophylactic UFH on angiogenic biomarkers. Data are shown as the regression coefficient and 95% CIs. The analysis was adjusted for maternal age, BMI > 25 kg/m², gestational age at blood sampling, and LDA intake. UFH, Unfractionated Heparin; CI, confidence interval; sFlt-1, soluble fms-like tyrosine kinase-1; PlGF, Placental Growth Factor

Dependent Variable	Unstandardized Coefficient for UFH group	95% CI	*P* value
sFlt-1 (pg/mL)	222.5	-128.5 to 573.4	0.21
PlGF (pg/mL)	31.4	-15.8 to 78.5	0.19
sFlt-1/PlGF	-0.70	-3.82 to 2.42	0.66

**Fig. 1:**
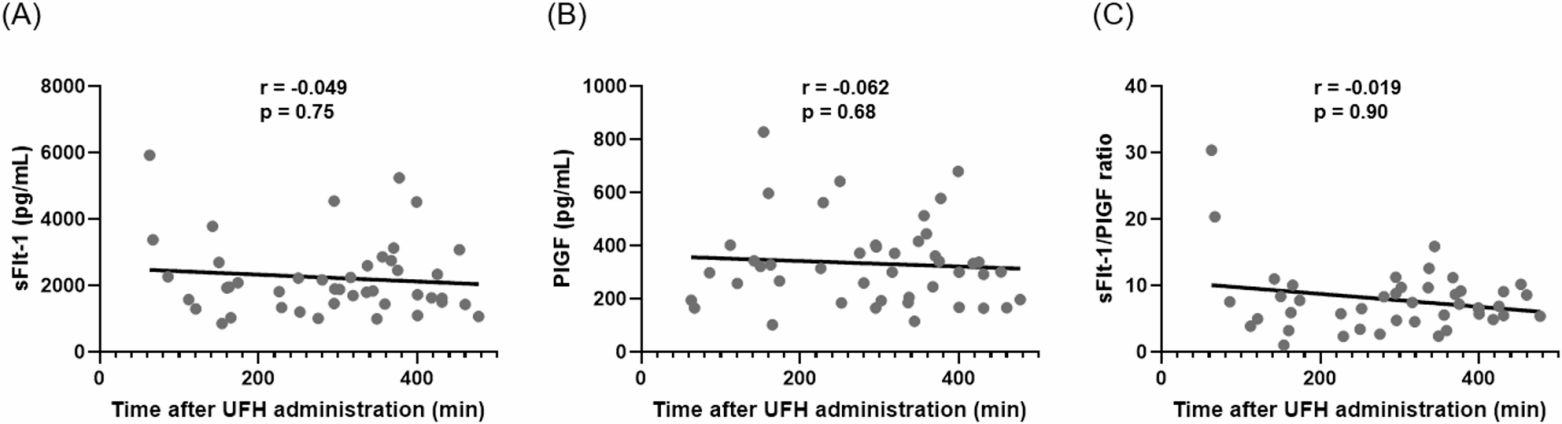
Correlations between time after UFH administration and biomarker levels. Scatter plots showing the relationship between the time from the last UFH administration (minutes) and (**A**) sFlt-1 concentration, (**B**) PlGF concentration, and (**C**) sFlt-1/PlGF ratio in the UFH group (*n* = 45). Spearman’s rank correlation coefficient (r) was used for statistical analysis. No significant correlation was observed with any of the items

Next, we evaluated the correlation between the time from the last UFH administration to blood collection and each biomarker value in the UFH group (*n* = 45) (Fig. 1). The time from UFH administration to blood collection was a median of 319 min (range 63–477 min) and included 4 samples collected within 120 min post-administration. The analysis revealed no statistically significant correlation between the time to blood collection and serum sFlt-1 concentration (*r*=-0.049, *p* = 0.75), PlGF concentration (*r*=-0.062, *p* = 0.68), or sFlt-1/PlGF ratio (*r* = 0.019, *p* = 0.90).

We evaluated the association between the duration of UFH therapy (from initiation to blood sampling) and biomarker levels. No significant correlations were observed between UFH duration and sFlt-1 (*r* = -0.12, *p* = 0.44), PlGF (*r* = -0.22, *p* = 0.15), or the sFlt-1/PlGF ratio (*r* = 0.12, *p* = 0.44).

Perinatal outcomes for both groups were also compared. Importantly, the incidence of PE was not significantly different between the UFH (1/45, 2.2%) and control (30/563, 5.3%; *p* > 0.99) groups. The UFH group had a significantly earlier gestational age at delivery (weeks+days: 38 + 2 vs. 38 + 6, *p* = 0.016). No significant differences in birth weight existed between the two groups (*p* = 0.14).

## Discussion

This study is the first to investigate the effect of prophylactic UFH anticoagulation therapy on the maternal serum sFlt-1/PlGF ratio and its time dependency. Our study produced two major findings. First, no statistically significant differences were observed in the levels of sFlt-1 or PlGF, or in the sFlt-1/PlGF ratio between the UFH and control groups. Second, within the UFH group, no correlation was identified between the sFlt-1/PlGF ratio and the time elapsed since UFH administration (Spearman’s *r* = 0.019, *p* = 0.90) within a clinically relevant sampling window of 63–477 min.

Previous studies have reported transient increases in serum sFlt-1 concentrations following heparin exposure. For example, subcutaneous LMWH (40 mg enoxaparin) increased serum sFlt-1 levels approximately 1.5-fold after 3 h in healthy volunteers [22], and intravenous UFH (5000 IU) used during hemodialysis caused an immediate rise in serum sFlt-1 levels that exceeded 50-fold [23, 24]. These findings suggest that blood sampling timing is a critical factor when interpreting the sFlt-1/PlGF ratio under subcutaneous LMWH or intravenous UFH administration [22–24]. By contrast, prophylactic subcutaneous UFH in this study did not demonstrate significant differences or time-dependent changes in the levels of serum sFlt-1 or PlGF, or the sFlt-1/PlGF ratio. Several factors may account for this discrepancy. First, subcutaneous UFH has a low and variable bioavailability (approximately 30%) [26–28], unlike the more stable bioavailability of subcutaneous LMWH (~ 90% [21]) or the complete bioavailability of intravenous UFH [23]. Second, the predominant dosage in our cohort was 10,000 IU/day, a standard prophylactic dose recommended for pregnant women with APS or a history of venous thromboembolism [17, 26, 27]. This dosage differs fundamentally from intravenous or therapeutic UFH regimens associated with marked sFlt-1 elevations. Taken together, these considerations suggest that the combination of subcutaneous administration and prophylactic dosing (5,000–10,000 IU/day) likely does not result in heparin concentrations sufficient to displace sFlt-1 from HSPG to a clinically meaningful extent. An in vitro study showed that UFH has a weaker ability to dissociate HSPG-bound sFlt-1 than LMWH [18], which may have less impact on circulating sFlt-1 levels, further supporting our interpretation.

The UFH group was comprised of pregnant women with APS or a history of thrombosis, who remain at an intrinsically high risk for PE even under prophylactic anticoagulation [13, 17]. In such patients, accurate PE risk assessment using the sFlt-1/PlGF ratio is particularly important. However, concerns have persisted that subcutaneous UFH might induce sFlt-1 elevation, similar to LMWH or intravenous UFH, thereby compromising interpretation of the biomarker. In this context, our findings are reassuring. Among women receiving prophylactic subcutaneous UFH, the sFlt-1/PlGF ratio was comparable to that of the control group, and no time-dependent influence of UFH was observed within the clinically relevant sampling window. Although blood sampling prior to UFH administration is theoretically ideal, in clinical practice the sFlt-1/PlGF ratio is measured at the time PE is suspected, particularly in outpatient settings, making it difficult to control the timing of UFH administration. Therefore, our findings may have practical relevance in real-world clinical settings.

The main limitation of this study is its cross-sectional, retrospective design, which does not allow assessment of temporal changes in the levels of sFlt-1 or PlGF after UFH administration within the same patient. Consequently, very early, transient post-dose fluctuations cannot be fully excluded, even though an apparent time-dependent pattern was not observed in the study cohort. The limited sample size and lack of serial measurements further restrict definitive conclusions regarding immediate post-dose dynamics. Prospective studies that incorporate repeated sampling before and after UFH administration are warranted to characterize the early temporal changes more precisely. A formal sample size calculation was not performed owing to the retrospective nature of the study. Additionally, as clinical use of the sFlt-1/PlGF ratio in Japan began in 2021, only cases that presented after its introduction were available for analysis, thereby limiting the sample size.　Additionally, our analysis was restricted to the mid-trimester. Since sFlt-1 and PlGF levels change dynamically throughout pregnancy, the potential influence of UFH could theoretically differ at other gestational ages. However, considering that heparin elevates sFlt-1 primarily through displacement from cell-surface HSPG—a fundamental mechanism unlikely to be gestational-age dependent—we anticipate that our findings are likely applicable to other phases of pregnancy as well. Furthermore, we assessed sFlt-1 and PlGF levels using Elecsys immunoassays (Roche Diagnostics) alone, and assays from other manufacturers were not evaluated. Therefore, the generalizability of these findings to other assay platforms remains to be determined.

## Conclusions

We found no association between prophylactic subcutaneous UFH administration and maternal sFlt-1/PlGF ratios. Moreover, the serum levels of sFlt-1 and PIGF were not correlated with the timing of administration. Given that women requiring anticoagulation are at high risk of PE, our findings provide evidence that this biomarker remains a valid tool for risk stratification in this population.

## Data Availability

The datasets used and/or analyzed during the current study are available from the corresponding author upon reasonable request.

## Abbreviations

APS: Antiphospholipid Syndrome
ART: Assisted Reproductive Technology
BMI: Body Mass Index
CH: Chronic Hypertension
CI: Confidence Interval
HSPG: Heparan Sulfate Proteoglycans
IQR: Interquartile Range
LDA: Low-Dose Aspirin
LMWH: Low-Molecular-Weight Heparin
PE: Preeclampsia
PlGF: Placental Growth Factor
sFlt-1: Soluble fms-like tyrosine kinase-1
UFH: Unfractionated Heparin

## References

[1] Chaiworapongsa T, Chaemsaithong P, Yeo L, Romero R. Pre-eclampsia part 1: current understanding of its pathophysiology. Nat Rev Nephrol. 2014;10:466–80. 10.1038/nrneph.2014.102. 25003615 10.1038/nrneph.2014.102 PMC5893150

[2] Stepan H, Hund M, Andraczek T. Combining biomarkers to predict pregnancy complications and redefine preeclampsia: the angiogenic-placental syndrome. Hypertension. 2020;75:918–26. 10.1161/HYPERTENSIONAHA.119.13763. 32063058 10.1161/HYPERTENSIONAHA.119.13763 PMC7098437

[3] Steegers EAP, von Dadelszen P, Duvekot JJ, Pijnenborg R. Pre-eclampsia. Lancet. 2010;376:631–44. 10.1016/S0140-6736(10)60279-6. 20598363 10.1016/S0140-6736(10)60279-6

[4] Maynard SE, Min JY, Merchan J, Lim KH, Li J, Mondal S, et al. Excess placental soluble fms-like tyrosine kinase 1 (sFlt1) may contribute to endothelial dysfunction, hypertension, and proteinuria in preeclampsia. J Clin Invest. 2003;111:649–58. 10.1172/JCI17189. 12618519 10.1172/JCI17189 PMC151901

[5] Zeisler H, Llurba E, Chantraine F, Vatish M, Staff AC, Sennström M, et al. Predictive value of the sFlt-1:PlGF ratio in women with suspected preeclampsia. N Engl J Med. 2016;374:13–22. 10.1056/NEJMoa1414838. 26735990 10.1056/NEJMoa1414838

[6] Bian X, Biswas A, Huang X, Lee KJ, Li TKT, Masuyama H, et al. Short-term prediction of adverse outcomes using the sFlt-1 (soluble fms-like tyrosine kinase 1)/PlGF (placental growth factor) ratio in Asian women with suspected preeclampsia. Hypertension. 2019;74:164–72. 10.1161/HYPERTENSIONAHA.119.12760. 31188674 10.1161/HYPERTENSIONAHA.119.12760 PMC6587370

[7] Zeisler H, Llurba E, Chantraine FJ, Vatish M, Staff AC, Sennström M, et al. Soluble fms-like tyrosine kinase-1 to placental growth factor ratio: ruling out pre-eclampsia for up to 4 weeks and value of retesting. Ultrasound Obstet Gynecol. 2019;53:367–75. 10.1002/uog.19178. 30014562 10.1002/uog.19178 PMC6590225

[8] Stepan H, Galindo A, Hund M, Schlembach D, Sillman J, Surbek D, et al. Clinical utility of sFlt-1 and PlGF in screening, prediction, diagnosis and monitoring of pre-eclampsia and fetal growth restriction. Ultrasound Obstet Gynecol. 2023;61:168–80. 10.1002/uog.26032. 35816445 10.1002/uog.26032

[9] Velegrakis A, Kouvidi E, Fragkiadaki P, Sifakis S. Predictive value of the sFlt-1/PlGF ratio in women with suspected preeclampsia: An update (Review). Int J Mol Med. 2023;52:89. 10.3892/ijmm.2023.5292. 37594116 10.3892/ijmm.2023.5292 PMC10500221

[10] Yusuf AM, Kahane A, Ray JG. First and second trimester serum sFlt-1/PlGF ratio and subsequent preeclampsia: A systematic review. J Obstet Gynaecol Can. 2018;40:618–26. 10.1016/j.jogc.2017.07.014. 28927814 10.1016/j.jogc.2017.07.014

[11] Kornacki J, Wender-Ozegowska E, Boroń D, Mantaj U, Wirstlein P, Gutaj P. sFlt-1/PlGF ratio in the prediction of preeclampsia in pregnant women with diabetic kidney disease. J Diabetes Res. 2025;2025:3987453. 10.1155/ jdr /3987453. 40521220 10.1155/jdr/3987453 PMC12165755

[12] Hamulyák EN, Scheres LJ, Marijnen MC, Goddijn M, Middeldorp S. Aspirin or heparin or both for improving pregnancy outcomes in women with persistent antiphospholipid antibodies and recurrent pregnancy loss. Cochrane Database Syst Rev. 2020;5:CD012852. 32358837 10.1002/14651858.CD012852.pub2 PMC7195627

[13] Depietri L, Veropalumbo MR, Leone MC, Ghirarduzzi A. Antiphospholipid syndrome: state of the art of clinical management. Cardiovasc Drugs Ther. 2023;39:385–404. 37572208 10.1007/s10557-023-07496-3

[14] Lim W, Crowther MA, Eikelboom JW. Management of antiphospholipid antibody syndrome: a systematic review. JAMA. 2006;295:1050–7. 16507806 10.1001/jama.295.9.1050

[15] Hamulyák EN, Scheres LJJ, Goddijn M, Middeldorp S. Antithrombotic therapy to prevent recurrent pregnancy loss in antiphospholipid syndrome-What is the evidence? J Thromb Haemost. 2021;19:1174–85. 33687789 10.1111/jth.15290 PMC8252114

[16] Middleton P, Shepherd E, Gomersall JC. Venous thromboembolism prophylaxis for women at risk during pregnancy and the early postnatal period. Cochrane Database Syst Rev. 2021;3:CD001689. 33779986 10.1002/14651858.CD001689.pub4 PMC8092635

[17] Tektonidou MG, Andreoli L, Limper M, Amoura Z, Cervera R, Costedoat-Chalumeau N, et al. EULAR recommendations for the management of antiphospholipid syndrome in adults. Ann Rheum Dis. 2019;78:1296–304. 31092409 10.1136/annrheumdis-2019-215213 PMC11034817

[18] Rosenberg VA, Buhimschi IA, Lockwood CJ, Paidas MJ, Dulay AT, Ramma W, et al. Heparin elevates circulating soluble fms-like tyrosine kinase-1 immunoreactivity in pregnant women receiving anticoagulation therapy. Circulation. 2011;124:2543–53. 22082677 10.1161/CIRCULATIONAHA.111.046821

[19] Sela S, Natanson-Yaron S, Zcharia E, Vlodavsky I, Yagel S, Keshet E. Local retention versus systemic release of soluble VEGF receptor-1 are mediated by heparin-binding and regulated by heparanase. Circ Res. 2011;108:1063–70. 21415391 10.1161/CIRCRESAHA.110.239665

[20] Park M, Lee ST. The fourth immunoglobulin-like loop in the extracellular domain of FLT-1, a VEGF receptor, includes a major heparin-binding site. Biochem Biophys Res Commun. 1999;264:730–4. 10544000 10.1006/bbrc.1999.1580

[21] Moore KH, Chapman H, George EM. Unfractionated heparin displaces sFlt-1 from the placental extracellular matrix. Biol Sex Differ. 2020;11:34. 32600401 10.1186/s13293-020-00311-w PMC7325113

[22] Hagmann H, Bossung V, Belaidi AA, Fridman A, Karumanchi SA, Thadhani R, et al. Low-molecular weight heparin increases circulating sFlt-1 levels and enhances urinary elimination. PLoS ONE. 2014;9:e85258. 24465515 10.1371/journal.pone.0085258 PMC3897409

[23] Searle J, Mockel M, Gwosc S, Datwyler SA, Qadri F, Albert GI, et al. Heparin strongly induces soluble fms-like tyrosine kinase 1 release in vivo and in vitro–brief report. Arterioscler Thromb Vasc Biol. 2011;31:2972–4. 21979436 10.1161/ATVBAHA.111.237784

[24] Morisawa H, Hirashima C, Sano M, Nagayama S, Takahashi H, Shirasuna K, et al. Difficulty of predicting early-onset super-imposed preeclampsia in pregnant women with hemodialysis due to diabetic nephropathy by serum levels of sFlt-1, PlGF, and sEng. CEN Case Rep. 2020;9:101–5. 31728843 10.1007/s13730-019-00435-y PMC7148397

[25] Ziakas PD, Pavlou M, Voulgarelis M. Heparin treatment in antiphospholipid syndrome with recurrent pregnancy loss: a systematic review and meta-analysis. Obstet Gynecol. 2010;115:1256–62. 20502298 10.1097/AOG.0b013e3181deba40

[26] Leffert L, Butwick A, Carvalho B, Arendt K, Bates SM, Friedman A, et al. The Society for Obstetric Anesthesia and Perinatology consensus statement on the anesthetic management of pregnant and postpartum women receiving thromboprophylaxis or higher dose anticoagulants. Anesth Analg. 2018;126:928–44. 29099429 10.1213/ANE.0000000000002530

[27] Hirsh J, Bauer KA, Donati MB, Gould M, Samama MM, Weitz JI. Parenteral anticoagulants: American College of Chest Physicians Evidence-Based Clinical Practice Guidelines (8th Edition). Chest. 2008;133(6) Suppl:141S-59S. 10.1378/chest.08-0689 18574264

[28] Garcia DA, Baglin TP, Weitz JI, Samama MM. American College of Chest Physicians Evidence-Based Clinical Practice Guidelines. Parenteral anticoagulants: antithrombotic Therapy and Prevention of Thrombosis, 9th ed: American College of Chest Physicians Evidence-Based Clinical Practice Guidelines. Chest. 2012;141(2) Suppl:e24S-43S. 10.1378/chest.11-2291 PMC3278070 22315264

[29] Nutescu EA, Spinler SA, Wittkowsky A, Dager WE. Low-molecular-weight heparins in renal impairment and obesity: available evidence and clinical practice recommendations across medical and surgical settings. Ann Pharmacother. 2009;43:1064–83. 19458109 10.1345/aph.1L194

[30] Magee LA, Brown MA, Hall DR, Gupte S, Hennessy A, Karumanchi SA, et al. The 2021 International Society for the Study of Hypertension in Pregnancy classification, diagnosis & management recommendations for international practice. Pregnancy Hypertens. 2022;27:148–69. 35066406 10.1016/j.preghy.2021.09.008

[31] Kanda Y. Investigation of the freely available easy-to-use software ‘EZR’ for medical statistics. Bone Marrow Transpl. 2013;48:452–8. 10.1038/bmt.2012.244 PMC3590441 23208313

[32] Tsiakkas A, Duvdevani N, Wright A, Wright D, Nicolaides KH. Serum soluble fms-like tyrosine kinase-1 in the three trimesters of pregnancy: effects of maternal characteristics and medical history. Ultrasound Obstet Gynecol. 2015;45:584 – 90. doi: 10.1002/uog.14817. Epub. PMID: 25678265. 10.1002/uog.14817 25678265

[33] Tsiakkas A, Duvdevani N, Wright A, Wright D, Nicolaides KH. Serum placental growth factor in the three trimesters of pregnancy: effects of maternal characteristics and medical history. Ultrasound Obstet Gynecol. 2015;45:591-8. 10.1002/uog.14811. Epub. PMID: 25653039. 10.1002/uog.14811 25653039

